# What is manipulation? A new definition

**DOI:** 10.1186/s12891-023-06298-w

**Published:** 2023-03-15

**Authors:** David W. Evans, Nicholas Lucas

**Affiliations:** 1 Centre of Precision Rehabilitation for Spinal Pain, School of Sport, Exercise and Rehabilitation Sciences, University of Birmingham, Birmingham, B15 2TT UK; 2 Research Centre, University College of Osteopathy, London, UK; 3 Private Practice, Sydney, Australia

**Keywords:** Manipulation, Mobilization, Adjustment, Manual therapy, Intervention, High velocity, Thrust, Cavitation, Definition

## Abstract

**Background:**

Definitions are important in healthcare. Unfortunately, problems can be found withall existing definitions of manipulation.

**Methods:**

This paper derives a set of eligibility criteria from prior definitions of manipulation to inform what should (and should not) be incorporated within a valid definition. These criteria were then used to select components from currently available empirical data to create a new definition.

**Results:**

The resulting definition of manipulation is: “*Separation (gapping) of opposing articular surfaces of a synovial joint, caused by a force applied perpendicularly to those articular surfaces, that results in cavitation within the synovial fluid of that joint.*” The corresponding definition for the *mechanical response* of a manipulation is: “*Separation (gapping) of opposing articular surfaces of a synovial joint that results in cavitation within the synovial fluid of that joint.*” In turn, the *action* of a manipulation can be defined as: “*A force applied perpendicularly to the articular surfaces.*”

**Conclusions:**

We believe these definitions to be valid (derived from and consistent with all available empirical data), complete (containing all necessary components), minimally sufficient (minimal redundancy, and sufficient to distinguish manipulation from other physical interventions), and robust (able to withstand important limitations embodied within sensible eligibility criteria). It is hoped that the simplicity and clarity of these definitions, and the transparency of their formation, will encourage their wide adoption in clinical, research, educational and professional settings.

## Introduction

Definitions are important in healthcare. Amongst other things, they facilitate diagnosis [1, 2], consistency of care [3], measurement of outcomes [4, 5], monitoring patient safety [6], education of clinicians [7], appropriate funding of care [8], and reproducible research [1, 2, 9]. It is safe to say that the manipulation of joints is no less in need of a valid definition than any other healthcare intervention. With this in mind, more than a decade ago [10], we constructed a list of necessary features, drawn from all available evidence at the time, that we believed should form the basis of a valid definition of manipulation (Table 1).

**Table 1:** Necessary features of manipulation (from Evans & Lucas 2010 [10])

***Action (that which the practitioner does to the recipient)***
A force is applied to the recipient
The line of action of this force is perpendicular to the articular surface of the affected joint
***Mechanical response (that which occurs within the recipient)***
The applied force creates motion at a joint
This joint motion includes articular surface separation
Cavitation occurs within the affected joint

In our 2010 paper [10], we stopped short of proposing a fully-fledged formal definition of manipulation in the hope that others might have joined us in completing this task. Our intention for involvement across multiple disciplines was that it would make any such definition more inclusive and acceptable. However, nobody did join us. Yet in the meantime, evidence supporting our position continued to grow (and still does) while problematic definitions (see Table 2) continued to be used across a diverse range of locations. We will therefore attempt to finish the task in this paper.

**Table 2:** Examples of existing definitions of manipulation

Source	Details	Definition
American Association of Colleges of Osteopathic Medicine, 2017 [11]	Professional organisation, USA	*“Therapeutic application of manual force”*
Sandoz, 1976 [12]	Expert opinion, Switzerland	*“A passive, manual manoeuvre during which an articular element is suddenly carried beyond the usual, physiological limit of movement without however exceeding the boundaries of anatomical integrity. The usual but not obligate characteristic of an adjustment is the thrust which is a brief, sudden and carefully dosed impulsion delivered at the end of the normal passive range of movement and which is usually accompanied by a cracking noise.”*
Nyberg, 1993 [13]	Expert opinion, USA	*“Thrust manipulation is the use of high velocity, low amplitude motion delivered at the end of the restricted physiologic limit of a joint’s range of motion*.”
Gatterman & Hansen 1994 [14]	Consensus of chiropractors, international	*“A manual procedure that involves a directed thrust to move a joint past the physiological range of motion, without exceeding the anatomical limit”*
Chartered Society of Physiotherapy, 2006 [15]	Professional organisation, UK	*“High velocity, low amplitude passive movements that are applied directly to the joint or through leverage”*
International Federation of Orthopaedic Manipulative Therapy, 2016 [16]	Professional organisation, international	“A passive, high velocity, low amplitude thrust applied to a joint complex within its anatomical limit* with the intent to restore optimal motion, function, and/or to reduce pain. *anatomical limit: Active and passive motion occurs within the range of motion of the joint complex and not beyond the joint’s anatomic limit.”
Government of Ontario, 1991 [17]	Primary legislation, Canada	*“Moving the joints of the spine beyond a person’s usual physiological range of motion using a fast low-amplitude thrust.”*
Parliament of New South Wales, 2001 [18]	Primary legislation, Australia	*“Spinal manipulation means the rapid application of a force (whether by manual or mechanical means) to any part of a person’s body that affects a joint or segment of the vertebral column.”*
McCarthy et al. 2015 [19]	Expert opinion, international	*“Spinal manipulation is the application of rapid movement to vertebral segments producing joint surface separation, transient sensory afferent input and reduction in perception of pain. Joint surface separation will commonly result in intra-articular cavitation that, in turn, is commonly accompanied with an audible pop. Post-manipulation reductions in pain perception are influenced by supraspinal mechanisms including expectation of benefit.”*

**Fig. 1:**
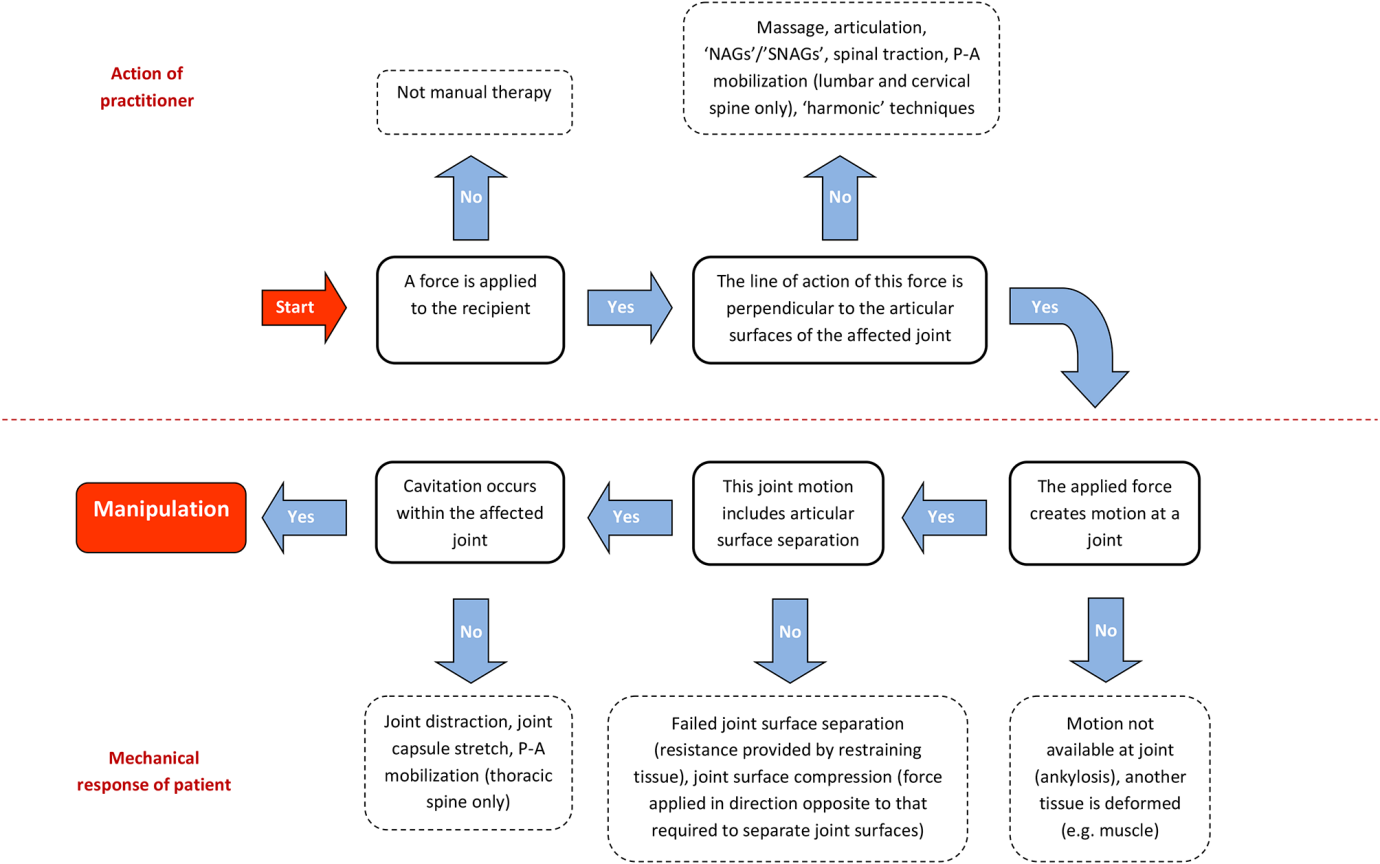
Causal pathway relating the necessary features of manipulation

In 2010 [10], we not only argued that the features listed in Table 1 were necessary; we also argued that these features were minimally sufficient, being the fewest number of features that collectively were sufficient in describing the characteristics of manipulation as it may occur in *any* synovial joint in the body. In this regard, it is reassuring that these necessary features fit neatly into a causal pathway without any obvious gaps (Fig. 1).

Existing definitions of manipulation (e.g., those in Table 2) clearly broaden the scope of components beyond those we suggested in 2010 [10]. Hence, before focusing on the individual components that should constitute a new definition of manipulation, consideration needs to be given to the *types* of components that should be included and excluded, which need to be selected in a systematic manner. This can be achieved by setting eligibility criteria. Doing so should then appropriately limit the scope and contents of any resulting definition.

## Eligibility of components for a definition of manipulation

For manipulation, we believe that an ‘intensional’ definition is required. An intensional definition incorporates all necessary and sufficient components, rather than creating an exhaustive list to cover every eventuality (an ‘extensional’ definition). Equally important, surplus or redundant components should be excluded. To begin this process, one can identify categories of components from previous definitions (Table 2) and then consider the relative merits of each category. Manipulation can also be compared to other healthcare interventions to inform the process.

### Consistency

Components of an intensional definition must be consistent; they must *always* occur when the defined event occurs. A corollary of this is that characteristics that occur some of the time (even *nearly* all of the time) cannot be considered defining attributes. An obvious example for manipulation is the magnitude of applied force over time, and the resulting accelerations and velocities of motion. Four force-time phases have been identified as *typically* occurring during manipulation of spinal joints [20–26]: the *orientation* (or *wind-up*) phase; the *preload* phase; the *thrust* (or *impulse*) phase; and the *resolution* phase. Notably, not all of these phases occur during spinal manipulation, even when all of the events depicted in Fig. 1 do [26–29]. Indeed, many clinicians are likely to have observed cavitation occurring during the low force magnitudes and low velocity motions that are typical of the wind-up phase. Additionally, low magnitude force and low velocity motion is the daily experience of millions of habitual ‘knuckle crackers’ around the world.

### Intention

Some previous definitions of manipulation [11, 16] have included the clinician’s intentions. For example, “*the intent to restore optimal motion, function, and/or to reduce pain*” [16]. Including intentions in a definition of any healthcare intervention is problematic given that history provides countless examples where good intentions alone were insufficient to achieve clinical benefit. It is totally plausible that an intervention delivered with therapeutic intent can lead to an adverse outcome, and manipulation is no exception here [30–32]. It is also conceivable that an intervention delivered with ill intent could lead to an unintended beneficial outcome. Furthermore, watching (and listening to) manipulation has recently found itself becoming a source of mass entertainment via social media and online video sharing platforms, with some viewers claiming to derive pleasure from the sensory experience. It is likely that many of these videos exhibit manipulations being delivered without any therapeutic intent at all. Irrespective of ethical questions arising as to whether manipulation should be used as a source of entertainment, each still qualifies as a manipulation. Intentions are therefore irrelevant and should not appear in any definition related to a healthcare intervention, including manipulation.

### Biological target

For any intervention to have therapeutic potential, it must be able to act upon a biological target [33]. The biological target must be an irreducible structure of the organism, through which one or more physiological (or psychological) effects can be initiated by the intervention [34]. Such targets should be mentioned explicitly in any definition of that intervention. For pharmaceutical interventions, a biological target is typically a tissue receptor (at which effects are usually either agonistic or antagonistic). For manipulation, there is ample evidence that the biological target is a synovial joint [10, 25, 35–42].

### Mechanical response

The necessary features listed in Table 1 were deliberately split into two components respectively called the *action* (that which the practitioner does to the recipient) and the *mechanical response* (that which occurs within the recipient) [10]. Another useful comparison can be made with pharmacology here. *Pharmacodynamics* is essentially ‘what the drug does to the body’, which will commence at the biological target. Accordingly, the *dynamics* of manipulation (i.e., ‘what the manipulation does to the body’) will begin with events occurring at a synovial joint. Hence, a definition of manipulation should certainly incorporate the events of the *mechanical response*, all of which occur within the synovial joint (Table 1).

### Universality

When applied to a class of phenomena, an intensional definition must apply fully to all members of the class [7]. If a synovial joint is the biological target of manipulation, then it follows that a definition sufficient for one synovial joint needs to be sufficient for any other, irrespective of its bodily location. Continuing the pharmaceutical analogy, if a particular receptor type was the biological target of a certain drug, the location of those receptors would not affect the defining properties of the drug, and the route of administration could be amended to target specific tissues as required.

In turn, the necessary characteristics of the *mechanical response* will be those consistently observed across multiple different synovial joints, both in the periphery and within the spine. Indeed, of the synovial joints to have been studied, the metacarpophalangeal (MCP) joint is by far the most common. Notably, the highest quality basic science research on joint manipulation originated not from clinicians interested in its therapeutic potential, but bioengineers interested in the phenomena of MCP joint ‘cracking’ [36, 37]. This seminal work has been the basis for the development of models of manipulation [12, 24, 43], upon which several definitions have then been constructed [44].

### Action

An intervention, whether in healthcare or elsewhere, suggests the occurrence of some form of *action*. As can be seen in Table 2, there is certainly a precedent for including *that which the practitioner does to the recipient* in definitions of manipulation. However, it could be argued that the components of the *mechanical response* depicted in Fig. 1 (i.e., *that which occurs within the recipient*) could alone sufficiently define a manipulation, and that those of the *action* would therefore be redundant in any such definition. Indeed, Newton’s first law of motion already requires that a (component of) applied force must be perpendicular to the articular surfaces of a joint if they are to separate. However, the converse is also true: it would be necessary to include components of the *action* if the components of the *mechanical response* were alone insufficient to define manipulation.

It turns out that the elements of the *mechanical response* may be achievable outside of the order in which they are depicted in Fig. 1. This is because manipulation is not the only means through which gas bubbles may appear in synovial joints. For example, bubbles can be introduced into the intra-articular space of a joint using a needle [45, 46]. This increased intra-articular volume of gas will then proceed to separate the joint surfaces. In this scenario, the causal sequence of events of the *mechanical response* depicted in Fig. 1 is effectively reversed. By explicitly incorporating the *action* into a definition of manipulation, the causal sequence depicted in Fig. 1 is preserved and potential misinterpretation is avoided. Hence, this is a reasonable justification for including the *action* in definitions of manipulation.

### Origins of action

Separate from intention, several prior definitions of manipulation have – at their root – explicitly described (and thereby limited) the origin of the *action* that initiates the intervention. Examples include, “*A passive, manual manoeuvre …*” [12], “*A manual procedure …*” [14], “*High velocity, low amplitude passive movements …*” [15], “*A passive, high velocity, low amplitude thrust …*” [16]. As can be seen in these examples, the terms ‘manual’ (i.e., via the hands) and ‘passive’ appear prominently and regularly. Limiting the origins of the *action* is problematic because its elements (described in Table 1 as a force applied to the recipient with its line of action being perpendicular to the articular surface of the joint) can be achieved without being either manual or passive. Indeed, it is entirely plausible (and easily demonstrable) that a machine or device could generate such a force, or that the recipient utilises self-generated forces alone to achieve the *action*. One of the definitions listed in Table 2 [18] partially acknowledges this by expanding its limitations with, “*Spinal manipulation means the rapid application of a force (whether by manual or mechanical means) …*”. However, a safer and more future-proof solution is to altogether avoid including the origins of the *action* in definitions of manipulation.

### Downstream effects

Previous definitions of manipulation have incorporated putative downstream consequences; for example, “*transient sensory afferent input*” [19] and “*supraspinal mechanisms including expectation of benefit*” [19]. The problem here is that any measured consequence of the *mechanical response* of a manipulation that might appear downstream from the biological target of the synovial joint (e.g., axonal conduction, synaptic transmission, descending modulation, perception, placebo responses, etc.) will act through physiological pathways that are not exclusive to manipulation and could feasibly be produced in some alternative way (e.g., electrical stimulation). Hence, these downstream physiological pathways can be shared by other interventions and are therefore surplus to the essential characteristics required to define manipulation. Consequently, they must fall outside of the scope of a definition of manipulation.

Downstream consequences of manipulation are also dependent upon the person being alive, and all that is bestowed by this. Yet, a manipulation performed on a cadaver must still constitute a manipulation if all necessary components are deemed to occur. Indeed, multiple important cadaveric studies of manipulation [21, 47–55] would be rendered invalid if this was not the case. This constraint would be the same if defining a surgical procedure (e.g., an appendectomy), which can clearly be performed to completion on a cadaver, and therefore seems just as appropriate for manipulation.

### Conditions

Some healthcare interventions, such as the extraction of a splinter or the relocation of a dislocated bone, can only be performed if a certain disease or pathological state is present. These are known as *necessary conditions* for the intervention. As such, the intervention itself must be defined by such a state. Notable by its absence in previous definitions of manipulation is the lack of reference to a specific disease or pathological state; although one definition [13] did impose the condition that manipulation is “*delivered at the end of the restricted physiologic limit of a joint’s range of motion*.”

If an intervention is considered potentially therapeutic for a specific disease or pathological state, these conditions are considered *indications* for its use. Conversely, if a specific disease or pathological state poses a risk for an intervention, these conditions are known as *contra-indications*. Incorporating indications and contra-indications into definitions of interventions can be problematic because they can be different for individual patients. For example, if spinal pain was incorporated into a definition of manipulation, how does one then deal with spinal pain that is caused by spinal malignancy, osteoporotic fractures, tuberculosis, or a cervical artery dissection? Such a definition would become *extensional*, requiring exhaustive lists that would require endless modification and caveats over time as new data arose. By instead using an intensional definition, the appropriateness of each incorporated necessary component of the intervention can be judged for every individual patient, and the need for such a list is avoided.

Since manipulation can be performed upon either a healthy or an unhealthy synovial joint (as countless training courses, social media videos, and studies on manipulation will attest), it is an intervention whose occurrence can be independent of health status, the presence or absence of disease, and pathological states. Hence, it should also be defined independently of such conditions.

### Outcomes

Previous definitions of manipulation have incorporated beneficial clinical outcomes, such as pain relief [16, 19]. However, an intervention can feasibly produce nil clinical benefit or even an adverse effect. Certainly, not every participant in every trial of manipulation has reported clinical improvements [56–59] and case studies reporting adverse events following manipulation do exist [30–32]. Thus, committing to a definition based upon beneficial outcomes would equally require us to define by these less attractive outcomes. In addition, models of manipulation [12, 24, 43, 44] have explicitly drawn upon data from MCP joints and cadaveric studies, in which no clinical outcome was either measured, sought or possible. If such models are accepted as representing manipulation, then clinical outcome must be outside of the scope of a definition.

In particular, the *mechanical response* of a manipulation must be separated out from the clinical outcome. In their discussion of definitions, McCarthy and colleagues [19] used the example of an Epley manoeuvre, in which a deliberate series of movements (i.e., the *action* of the clinician) led to a *mechanical response* (which was reliant upon the presence of gravitational forces to act upon the vestibular apparatus). In turn, this *action* and consequential *mechanical response* can purportedly lead to canalith repositioning, but this mechanistic pathway is only available if canalith *malposition* is a pre-existing pathological state within the recipient. In other words, both gravitational forces and canalith malposition are necessary conditions for this mechanistic pathway to exist. Finally, the recipient may or may not result in a beneficial clinical outcome (and this may or may not be related to the above mechanistic pathway). From randomised controlled trials of Epley manoeuvres for the canonical condition of benign paroxysmal positional vertigo [60], it is clear that the clinical outcome is not always beneficial to the recipient. Nevertheless, every attempt will have still qualified as an Epley manoeuvre (the movements were performed, and gravitational forces were present), illustrating the problem with attempts to incorporate clinical outcomes into definitions of healthcare interventions.

### Taxonomic consistency

A useful definition of manipulation must fit within a wider taxonomy of physical interventions, and in particular those within the domain of manual therapy. Within such a taxonomy, different interventions should be distinguishable and mutually exclusive of one another by readily measurable factors. By historical convention (and best available empirical data) these factors should be mechanical in nature. By contrast, the clinical outcomes of such physical interventions appear to be very similar [56], which is not helpful if outcome is used to distinguish one intervention from another. This further reinforces why clinical outcomes should be separated from the respective *action* and *mechanical response* from which they would originate.

As we stated in 2010 [10], the causal pathway depicted in Fig. 1 can be used as the starting point for a wider taxonomy for physical interventions based entirely on empirical characteristics. Indeed, this wider taxonomy has yet to be fully developed to include all physical interventions; we still hope that others will join in with that particular pursuit.

### Summary of eligibility criteria

Collectively, the eligibility criteria set out above and summarised in Table 3 can serve as a series of ‘tests’ that any proposed definition of manipulation will need to withstand. It is noteworthy that none of the existing definitions listed in Table 2, nor any others that we have seen elsewhere, fully comply with these criteria.

**Table 3:** Eligibility criteria for components of a new definition of manipulation

Component	Criterion	Include/exclude
Consistency	Manipulation should not be defined by characteristics that occur inconsistently	Exclude
Intention	Intentions should not appear within a definition of any healthcare intervention (including manipulation)	Exclude
Biological target	The biological target of a manipulation is a synovial joint and should be incorporated within a definition	Include
Mechanical response	A definition of manipulation should incorporate events that consistently occur in and around affected synovial joint(s)	Include
Universality	A definition of manipulation that is sufficient for one synovial joint should be sufficient for any other	Include
Action	A definition of manipulation should incorporate the active components that are necessary to create the mechanical response	Include
Origins of action	A definition of manipulation should not place unnecessary limits upon the origins of the action	Exclude
Downstream effects	Manipulation should not be defined by downstream effects on shared physiological pathways	Exclude
Conditions	The occurrence of manipulation should not be defined by conditions of health, disease, or pathology	Exclude
Outcomes	Healthcare interventions (including manipulation) should not be defined by clinical outcomes	Exclude
Taxonomic consistency	A definition of manipulation should be taxonomically consistent with other (manual therapy) interventions	Include

## Components of a new definition

Several existing definitions (e.g., Table 2) incorporate some unusual terms. The term *thrust*, for example, is used in the colloquial sense and is therefore inappropriate for a formal definition [44]. Thrust is a reaction force (i.e., a force that acts in the opposite direction to the line of action of an applied force) described quantitatively by Newton’s third law of motion, which states that all forces between two objects exist in equal magnitude and opposite direction. Thrust is produced by a rocket’s engine when it rapidly expels the mass of its burned fuel in one direction, which simultaneously creates a reaction force that propels the rocket in the opposite direction. If the term must be used in the context of manipulation, thrust is technically the reaction force from the recipient to the practitioner, not the other way around.

Previous definitions have attributed importance to the velocity of joint motion generated during manipulation. Specifically, *high-velocity* motion is alluded to in nearly all previous definitions of manipulation (including those listed in Table 2), expressed as “*rapid movement*”, “*rapid application*”, “*sudden*”, “*fast*”, “*high velocity*”, etc. The ‘critical’ velocity of joint motion required to initiate cavitation within synovial fluid is likely to be very low [10, 61], which is consistent with studies of MCP joint cavitation [35–37, 40–42, 62, 63]. Additionally, multiple studies have described cavitation occurring during spinal manipulation in the absence of high velocity motion [27, 28]. This variability of the velocity of motion during manipulation is the primary reason why it cannot be considered a defining attribute. This also means that ‘*high velocity-low amplitude thrust*’ or similar misnomers should be avoided.

**Fig. 2:**
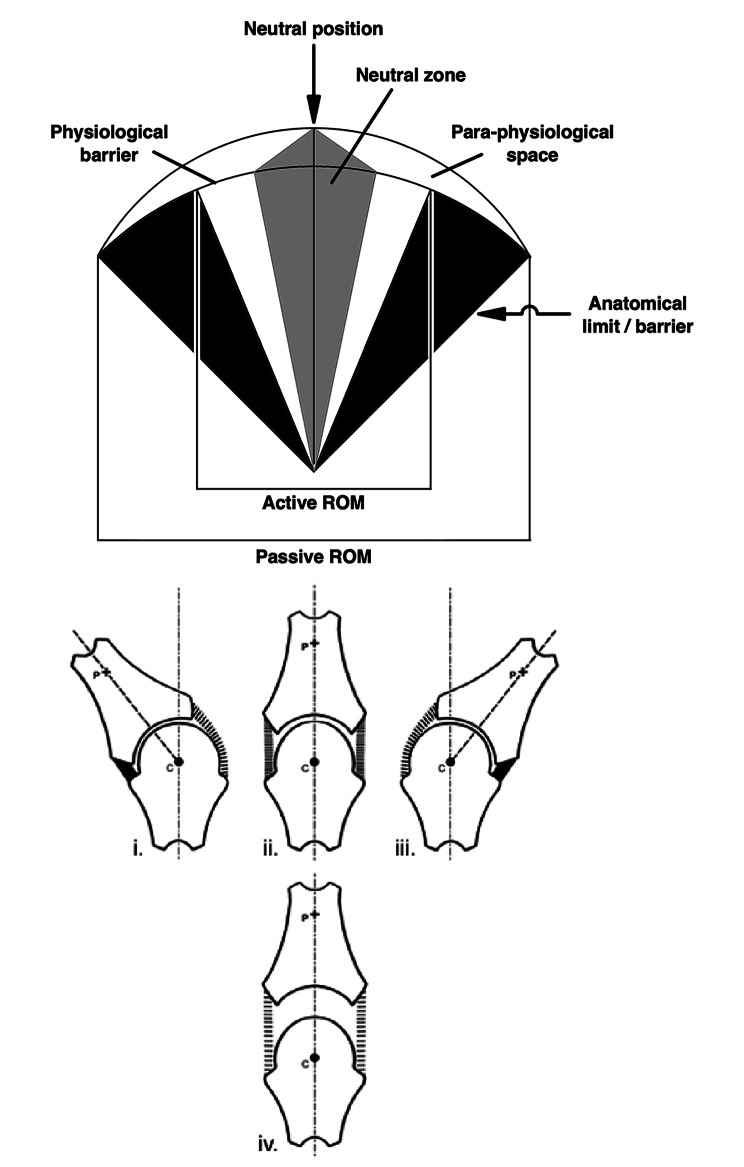
The corrected model of joint manipulation. Based on Evans & Breen 2006 [24]. Reproduced from Evans 2022 [44]

As has been described in detail elsewhere [24, 43, 44], several of the unusual terms within previous definitions (e.g., *physiological range of motion*; *physiological limit of movement*; *anatomical limit*) originate from the influential but ultimately flawed model of Sandoz [12]. Notably, most existing definitions are based upon this flawed model. A corrected version of this model, consistent with all available empirical data, was published in 2006 [24]. Importantly, this corrected model makes predictions that are consistent with all available empirical data and are also reassuring from a safety point of view [44]. For the convenience of readers, this corrected model is reproduced in Fig. 2.

As per Sandoz’s original model [12], Fig. 2 depicts a two-dimensional representation of the motion of a single synovial joint. Beneath this are partitioned diagrams of the same synovial joint in different configurations. This joint is symmetrical, and therefore idealised, resulting in a perfectly arc-shaped path drawn by the arbitrarily placed fixed point (*p*) during its rotational motion around a static centre of rotation (*c*). As guided by the partitioned diagrams, the relationship between the centre of rotation (*c*) and the fixed point (*p*) dictate both the correct location and extent of the *para-physiological space* [24, 44]. Unlike Sandoz’s original version [12], the *para-physiological space* resides upon the upper border of the arc depicting the rotational range of motion of the joint. While the joint surfaces are in contact, the *para-physiological space* has no area in this two-dimensional representation (nor volume in a real three-dimensional joint). The space is therefore a *potential* space, akin to that of the pleura, only becoming real and apparent when the surfaces separate [44].

Once familiarised with the corrected model of manipulation in Fig. 2, it is worth re-reading the existing definitions listed in Table 2 to see how far removed they are from this model (and the data from which it was built). Whilst not all statements that constitute the definitions in Table 2 are outright false in light of the corrected model, they nearly all miss the quintessential kinematic component of manipulation (joint surface gapping) and all include redundant components (e.g., high velocity motion, clinical outcomes, intention, etc.). At this point, we can be justified for asking, ‘how should manipulation be defined?’

## A new definition of manipulation

Given the eligibility criteria and corrected model presented above, and the individual components from Table 1 that were considered at length in 2010 [10], we propose that manipulation should be defined as:

***Definition 1:*** *Separation (gapping) of opposing articular surfaces of a synovial joint, caused by a force applied perpendicularly to those articular surfaces, that results in cavitation within the synovial fluid of that joint.*

In turn, a definition for the *mechanical response* can be constructed by simply removing the text between the commas in Definition 1:

***Definition 2:*** *Separation (gapping) of opposing articular surfaces of a synovial joint that results in cavitation within the synovial fluid of that joint.*

Lastly, the *action* can be defined using the text between the commas in Definition 1:

***Definition 3:*** *A force applied perpendicularly to the articular surfaces.*

We believe that Definition 1 encompasses *all* necessary components of manipulation [10], as listed in Table 1. We also believe that Definition 1 possesses minimal redundancy. It is deliberately phrased in a manner that requires little explanation beyond an understanding of the individual words from which it is constructed. The extracted definitions of the *mechanical response* (Definition 2) and *action* (Definition 3) are not required for Definition 1 to stand alone but should be useful when practising, teaching, researching, and evaluating manipulation.

We argued above that both *action* and *mechanical response* should be included within a definition of manipulation. In line with our eligibility criteria, we have placed no restrictions on the origins of the force comprising the *action* (Definition 3); just that its line of action (or at least a component of it) is perpendicular to the articular surfaces of the joint [10]. Hence, we have avoided a preamble, such as, “*A manual procedure that involves* …” or “*A passive, manual manoeuvre during which* …”.

We have intentionally used the terms “*caused by* …” and “*results in* …” within Definition 1 to ensure the sequence of events described in Fig. 1 is preserved. In particular, the *mechanical response* (Definition 2) deliberately describes a causal pathway that occurs within the recipient, beginning with the separation of articular surfaces of a joint, which then results in the cavitation event within the synovial fluid of that joint; a sequence of events that has been demonstrated multiple times in mutually supporting independent studies [35–37, 39–41, 62]. Notably, the *mechanical response* (Definition 2) mentions nothing of *low-amplitude displacement*, nor any criterion relating to *ranges of motion*, *physiological limits*, *anatomical limits*, *boundaries*, *barriers*, or *tissue damage*, which we believe to be surplus to requirements for a definition, even though some of these concepts were retained in the corrected model of manipulation (Fig. 2).

In addition to the earlier arguments put forward, the explicit inclusion of the *action* (Definition 3) within Definition 1 should serve as a useful guide for clinicians in terms of what is – and what is not – required to create the *mechanical response* during the delivery of manipulation. Indeed, notably absent from the *action* (Definition 3) is any mention of *high-velocity motion*, which is alluded to in nearly all previous definitions of manipulation.

The occurrence of all components of Definition 1 will constitute a manipulation. A requirement for consistency commits one to define by the occurrence of a specific set of events. As such, if a clinician attempts to perform a manipulation and one or more of the necessary components do not occur, this will *not* constitute a manipulation but could instead be described as an ‘attempted’ manipulation. If all necessary components apart from cavitation occur, this would constitute ‘joint distraction’ (Fig. 1). Equally, if a clinician is not attempting to manipulate a joint, and yet all necessary components (including cavitation) occur, then this would constitute a manipulation but could be described as an ‘unintended’ manipulation. It is worth noting that at no point are we claiming that the occurrence of cavitation confers additional downstream effects or clinical benefits. It may or may not do so. We are instead arguing that the occurrence of manipulation is separated from both downstream effects and clinical outcomes so that their putative relationships can be accurately described and fairly evaluated.

Importantly, although downstream effects that might be specific to manipulation are not incorporated within the above definitions, such effects should be entirely attributable to the *mechanical response* (Definition 2), all components of which are measurable and verifiable [25, 28, 38]. This will allow one to distinguish between these and any ‘non-specific’ effects that might occur during the preparation and delivery of manipulation. The importance of this distinction is that any mechanistic pathways that are necessary for therapeutic mechanisms of action and clinical benefit should be more easily identified.

We are aware that some might be uncertain as to how these new definitions could and should be used. We believe that these new definitions will allow manipulation to be more easily taught to students, better studied by researchers, more fairly evaluated by guideline panellists, and that clinicians will be better able to decide upon indications and contra-indications for individual patients.

## Conclusion

This paper presents a new definition of manipulation, which we believe to be valid (derived from and consistent with all available empirical data), complete (containing all necessary components), minimally sufficient (minimal redundancy, and sufficient to distinguish manipulation from other physical interventions), and robust (able to withstand important limitations embodied within sensible eligibility criteria). Corresponding definitions for the *action* and *mechanical response* are also provided. It is hoped that the simplicity and clarity of these definitions, and the transparency of their formation, will encourage their wide adoption in clinical, research, educational and professional settings.

## Data Availability

Not applicable.
